# A Genetic Approach to Spanish Populations of the Threatened *Austropotamobius italicus* Located at Three Different Scenarios

**DOI:** 10.1100/2012/975930

**Published:** 2012-05-03

**Authors:** Beatriz Matallanas, Carmen Callejas, M. Dolores Ochando

**Affiliations:** Departamento de Genética, Facultad de Ciencias Biológicas, Universidad Complutense de Madrid, C/José Antonio Novais 2, 28040 Madrid, Spain

## Abstract

Spanish freshwater ecosystems are suffering great modification and some macroinvertebrates like *Austropotamobius italicus*, the white-clawed crayfish, are threatened. This species was once widely distributed in Spain, but its populations have shown a very strong decline over the last thirty years, due to different factors. Three Spanish populations of this crayfish—from different scenarios—were analysed with nuclear (microsatellites) and mitochondrial markers (*COI* and *16S* rDNA). Data analyses reveal the existence of four haplotypes at mitochondrial level and polymorphism for four microsatellite loci. Despite this genetic variability, bottlenecks were detected in the two natural Spanish populations tested. In addition, the distribution of the mitochondrial haplotypes and SSR alleles show a similar geographic pattern and the genetic differentiation between these samples is mainly due to genetic drift. Given the current risk status of the species across its range, this diversity offers some hope for the species from a management point of view.

## 1. Introduction

Spain is the country with the greatest biodiversity of Europe, with around 80,000 catalogued taxa. The maritime barrier of the Mediterranean, the land barrier of the Pyrenees in the North, and the country's orographic and climate peculiarities, invest it with unique biogeographic characteristics. Therefore, the country's large number of endemic—specially freshwater—species makes it a biodiversity hot spot [1].

At present, Spanish freshwater ecosystems are suffering great modification at the hands of climate change, environmental degradation, habitat fragmentation, the rise in human demand for water, and a range of human activities. Together, these factors have contributed to a notable increase in the size of Spain's arid and semiarid regions, and to changes in its biodiversity [2].

In 2008 more than 80% of Spanish endemisms were reported to suffer some level of threat in the IUCN Red List. At present, the assessments for some of these species have got worse. Among the macroinvertebrates, the crayfish *Austropotamobius italicus* was listed as vulnerable in 2008, but in 2010 it has been categorized as endangered [3].

*Austropotamobius italicus* was once a cornerstone of Iberian freshwater ecosystems with large populations widely distributed throughout most of the country's limestone basins. Indeed, it was absent in the more western areas, the highest mountain ranges, and the subdesert areas of the southeast and River Ebro valley. The dramatic decline in its numbers all over its Spanish range is the result of a combination of the factors mentioned above, as well as of the introduction of exotic crayfish species and the related spread of crayfish plague (caused by the fungus *Aphanomyces astaci*). As a consequence, only around 1000 small populations now remain in Spain (Alonso, pers. com.) occupying marginal areas or short stretches of watercourses usually isolated from the main river systems [4].

At present, restoration programs are based mainly on translocation of individuals from other natural or farmed populations and are limited by the low number and abundance of existing populations. Besides, it should be taken into account that availability of individuals for restocking purposes needs to be substantially increased by either traditional hatcheries or extensive ponds [5]. These action plans consider several factors such as the risk of transmission of crayfish plague, the risk of survival when establishing new populations, the characteristics of water bodies to be restored, or the distribution of exotic species in those areas [5, 6].

Notwithstanding, a major goal of such programs should also preserve genetic variability—the basis for viability and future evolution of populations [7, 8]. Indeed, knowledge of the levels and patterns of distribution of the genetic diversity is critical when making conservation management decisions. In other words, effective long-term conservation planning must incorporate genetic information [9] because the loss of genetic variation and inbreeding depression put wildlife populations at an increased risk [10].

In this context, our group is conducting a comprehensive study on the genetic variation and its distribution in *Austropotamobius italicus* populations from Spain. Our previous survey, by random amplified polymorphic DNA (RAPD) fingerprinting, detected a certain degree of polymorphism in some of the populations tested [11].

Three molecular markers were used in the present study, two of them mitochondrial and the other one, nuclear. This approach combines the advantages of both methods. It is clear that the mitochondrial genome of animals is an excellent target for genetic analysis because of its lack of introns, its limited exposure to recombination, and its haploid mode of inheritance [12]. Mitochondrial DNA (mtDNA) has proved to be powerful for genealogical and evolutionary studies of animal populations. Otherwise, microsatellite loci are highly polymorphic markers, distributed throughout the nuclear genome and generally not linked to loci under strong selection [13]. These codominant markers have revealed substantial variation in species with low variability in other nuclear markers [14] and have been used to study genetic differentiation among closely related populations [15].

Taking into account all above, our aim was to study the genetic variability of three Spanish populations of white-clawed crayfish belonging to three different scenarios—protected area, crashed population, and hatchery.

## 2. Material and Methods

### 2.1. Samples

A total of 45 individuals of *Austropotamobius italicus* were collected from three different populations (Table 1). One of them, located in a protected area: NAV, a native population. A second population, RIL, from a crayfish hatchery, maintained with a high effective number. Thirdly, GRA also a native population whose number crashed during the 1990s due to several pathologies.

For each population ten individuals were studied employing two mitochondrial markers: cytochrome oxidase subunit I (*COI*) and *16S* rDNA gene. For SSR analysis fifteen individuals from each of the three populations sampled were used.

### 2.2. DNA Isolation, Amplification, and Sequencing

Genomic DNA was extracted from 20–50 mg of claw muscle or periopod tissues (without killing the animal) using the DNeasy Blood and Tissue Kit from Qiagen (Valencia, CA, USA) and resuspended in Tris-EDTA (10 mM; 1 mM; pH 8.0).

DNA concentration and purity were estimated by absorbance at 260–280 nm in a NanoDrop ND-100 (NanoDrop Technologies, Wilmintong, USA) spectrophotometer. Its integrity was verified by 0.8% agarose gels in Tris-EDTA buffer (10 mM; 1 mM; pH8). Gels were stained with ethidium bromide (1 *μ*g/mL) and visualized with UV light transilluminator. All the polymerase chain reactions (PCR, [16]) were carried out using a Lab Cycler (SensoQuest, Göttingen, Germany).

A fragment from the mtDNA *COI* gene was amplified in a final volume of 50 *μ*L with 25 ng of total DNA, 1x reaction buffer, 2 mM MgSO_4_, 200 *μ*M of each dNTP, 15 *μ*g of BSA, 1 *μ*M of each primer, and 1 U of Vent DNA polymerase (New England Biolabs, Ipswich, MA, USA). The primers used were *COI* Scylla [17] and LCO [18]. The optimal PCR programme include an initial denaturation step of 94°C for 5 min followed by 44 cycles of 94°C for 45 s, 53°C for 1 min, and 72°C for 1 min 30 s, and a final extension step of 72°C for 10 min.

The selective amplification of a segment of the mtDNA *16S* rDNA was performed with the primers 1472 and Tor12sc [19], applying the following PCR conditions: an initial denaturation step of 95°C for 2 min followed by 9 cycles of 95°C for 25 s, 57°C for 30 s, and 72°C for 150 s, then 29 cycles of 95°C for 35 s, 54°C for 30 s, and 72°C for 150 s, finally an extension step of 72°C for 10 min. Each reaction, with a final volume of 50 *μ*L, contained 25 ng of total DNA, 1x reaction buffer, 2.2 mM MgCl_2_, 200 *μ*M of each dNTP, 330 *μ*g of BSA, 0.44 *μ*M of each primer, and 1 U of AmpliTaq DNA polymerase (Applied Biosystems, USA).

Double-stranded amplified products for both mitochondrial markers were purified with the High Pure PCR Product Purification Kit (Boehringer-Manheim) and used as templates for sequencing reactions. These reactions were carried out with the “BIG Dye Terminator Cycle Sequencing Ready Reaction Kit” (Applied Biosystems, Inc., USA) on a 3730 DNA Analyzer (Applied Biosystems, Inc., USA), using the primers employed for the amplification step, at the Genomic Unit of The Complutense University of Madrid.

The SSR study included five loci. All primers used were developed by Gouin et al.: Ap1, Ap2, Ap3, Ap5 reverse, Ap6 [20], and Ap5 forward [21]. SSR loci were amplified using a forward labelled primer with one of the Applied Biosystems fluorochromes 6-FAM, PET or VIC.

QIAGEN Multiplex PCR kit (Qiagen, Hilden, Germany) was used to amplify the Ap1, Ap2, Ap3, and Ap6 SSR loci. Reaction with a final volume of 6.5 *μ*L contained 5 ng of total DNA, 3.25 *μ*L of 2x QIAGEN Multiplex PCR Master Mix, 0.1 *μ*M of Ap1 and Ap6 primers, and 1 *μ*M of Ap2 and Ap3 primers. Amplification conditions were 95°C for 10 min followed by 36 cycles of 94°C for 30 s, 62°C for 60 s, and 72°C for 60 s, with an extension final step of 72°C for 10 min. Ap5 locus was amplified in a final volume of 6.5 *μ*L with 35 ng of total DNA, 1x reaction buffer, 1.9 mM MgCl_2_, 8 *μ*M of each dNTP, 0.2 *μ*M of forward labelled primer, 0.4 *μ*M of reverse primer, and 0.5 U of AmpliTaq DNA polymerase (Applied Biosystems, USA). The optimal PCR programme for this locus include an initial denaturation step of 94°C for 10 min followed by 15 cycles of 94°C for 30 s, 55°C for 35 s, and 72°C for 50 s, then 25 cycles of 94°C for 30 s, 60°C for 35 s and 72°C for 50 s, finally an extension step of 72°C for 10 min.

PCR products were run with the internal size standard GeneScan 500 LIZ (Applied Biosystems, USA) on a 3730 DNA Analyser (Applied Biosystems, USA). Allele size was determined through Peak Scanner Software v1.0 (Applied Biosystems, USA).

### 2.3. mtDNA Alignment and Sequence Analysis

The nucleotide sequences of the mitochondrial DNA were aligned using CLUSTAL W software [22] and edited with BioEdit v 7.0.9.0 [23]. After alignment and edition, amplified fragments, *16S* (1317 bp) and *COI* (1184 bp), were also used together to obtain a single sequence of 2501 bp length. The genetic diversity estimates (haplotype diversity, H; nucleotide diversity, *π*; number of segregating sites, S) were calculated using DnaSP v 5.10.01 programme [24].

*F*_ST_ pairwise genetic distances [25], which quantify how genetic diversity is partitioned within and between populations, and gene flow (Nm), were estimated through DnaSP v 5.10.01 software package [24]. Principal component analysis (PCA) [26] was performing using NTSYSpc v2.10q software package [27] to visualize the grouping populations. Finally, haplotype frequencies for each mitochondrial gene were geographically depicted for each population using PhyloGeoViz v 2.4.4 [28].

### 2.4. Microsatellite Analysis

Genetic diversity was quantified as the mean number of observed alleles per locus (*A*), effective number of alleles per locus (*n*_*e*_), the observed heterozygosity (*H*_*o*_) and the Hardy-Weinberg expected heterozygosity (*H*_*e*_) [29] through Popgene software [30]. Genepop v4 [31] was employed to estimate deviations from Hardy-Weinberg equilibrium across populations and across loci using the Markov chain method (10000 iterations). The software also tested the linkage disequilibrium across all populations and estimated the inbreeding coefficient *F*_IS_ within each population by the Weir and Cockerham [32] method.

To assess the effects of genetic drift and mutation in the structure of these populations, two statistics of genetic differentiation, *F*_ST_ [32] and Rho_ST_ [33], were calculated (Genepop software). *R*-statistics were expected to be larger than *F*-statistics when stepwise-like mutations have contributed to population differentiation [34]. Otherwise, if both statistics are similar, genetic drift is considered the main force for genetic differentiation. Wilcoxon's signed ranked test was performed to assess differences between *F*_ST_ and Rho_ST_ estimates.

Genetic structure of populations was inferred using the model-based clustering algorithms implemented in STRUCTURE v2.0 [35]. For parameter estimations, the admixture model with correlated allele frequencies was used (with 100000 MCMC iterations of burn-in length and 100000 after-burning repetitions).

In order to analyse the homogeneity of samples, a correspondence analysis (CA)—on the matrix of allele counts per sample, both at the population and the individual levels—was conducted using Genetix v4.05.2 software [36]. This technique is especially useful when the number of available loci is limited.

## 3. Results

### 3.1. mtDNA Analysis

A 2501 bp (1184-nucleotide sequence from *COI* gene and 1317 bp from *16S* rDNA gene) fragment was obtained from 30 individuals. Sequence analysis revealed four single nucleotide polymorphisms (SNPs), three of them informative under parsimony (Table 2). Four haplotypes were identified, three at high or intermediate frequencies (Haplotypes 1–3) and the remaining one (Hap_4), at low frequency. Likewise, Haplotypes 1 and 2, both differing in a transition in position 1536, account for 80% of individuals (Table 2).

Regarding the populations, NAV and RIL show genetic diversity at mtDNA gene level since two different haplotypes were detected in each sample (Hap_1 and Hap_3 in RIL population and Hap_2 and Hap_4 in NAV sample). The highest haplotype and nucleotide diversity were found in the farmed population (RIL) (Table 1).

As a whole, *F*_ST_ value revealed significant population differentiation at the mtDNA level (*F*_ST_ = 0.8547). The inferred Nm value was 0.09 that reduces to 0.03 when only the two natural populations are taking into account. The highest *F*_ST_ genetic distances were found between comparisons of NAV population and the other two samples (*F*_ST  NAV-GRA_ = 0.952, *F*_ST  NAV-RIL_ = 0.855).

**Figure 1:**
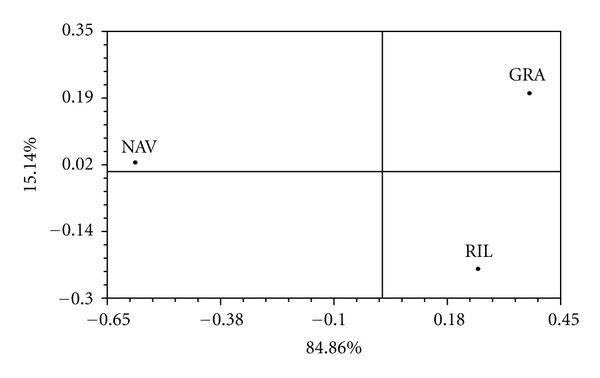
Results of the PCA analysis based on mtDNA sequences from three samples of *A. italicus*. Eigenvalues for each principal component are listed besides each axis.

Relationships among these three populations were visualized by the principal component analysis (Figure 1). The first PCA axis explains 84.86% of variance and reveals two well-separated groups: NAV (mainly Hap_2) and, GRA and RIL (mostly Hap_1). The second PCA axis explains 15.14% and disjoined populations with Hap_1 into two different groups: GRA (Hap_1) and RIL (Haplotypes 1 and 3).

**Figure 2:**
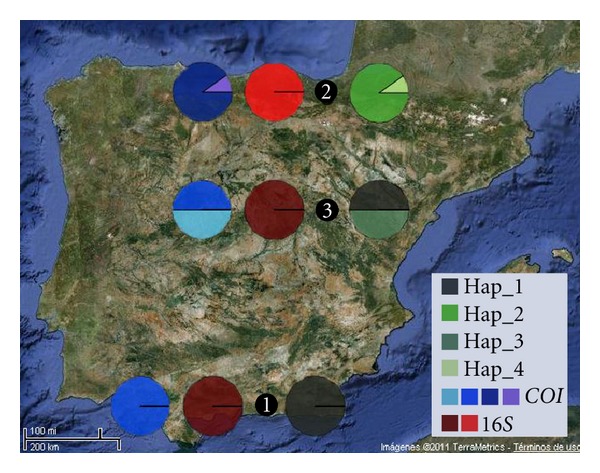
Geographic localization and genetic composition of 30 individuals from 3 different populations. Each population is shown as 3 pie charts representing membership proportions. In blue series: *COI* mtDNA gene, red series: *16S* mtDNA gene and green series as total mtDNA.

As shown in Figure 2, haplotypes found were not evenly distributed across samples. At *COI* level, GRA and RIL samples shared one of the haplotypes found, although GRA was monomorphic whereas RIL also presented a private haplotype. In addition, NAV population held two different and exclusive haplotypes. Nonetheless, only two different groups were observed at rDNA *16S* gene, since a single mutation separated the two haplotypes found. Thus, GRA and RIL shared the same haplotype while NAV sample held the other one.

### 3.2. Microsatellite Analysis

A total of 45 individuals were analysed through five SSR loci. Four of which were polymorphic—Ap1, Ap2, Ap3, and Ap6—whereas locus Ap5 was monomorphic.

**Figure 3:**
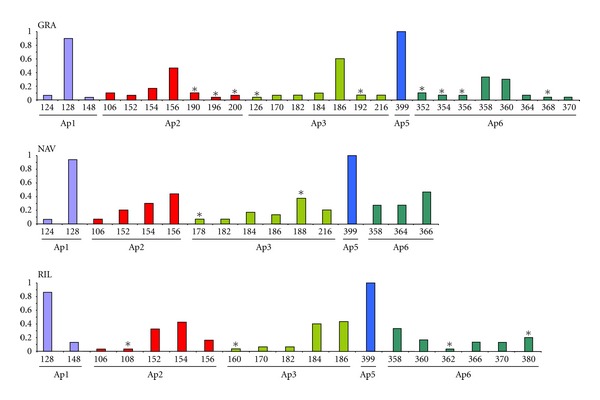
Allele frequencies in the populations of *A. italicus* for each microsatellite locus. Asterisk indicates private alleles.

The total number of alleles detected for the three Spanish populations was 33. All samples had private alleles—GRA: Ap2: 190, 196, and 200; Ap3: 126, and 192; Ap6: 352, 354, 356 and 368. NAV: Ap3: 178, and 188. RIL: Ap2: 108; Ap3: 150; Ap6: 362 and 380—usually at low frequencies. The number of alleles per locus ranged from 1 (locus Ap5) to 8 (locus Ap6) and their frequencies are shown in Figure 3.

Parameters of genetic diversity are displayed in Table 3. GRA population had the highest values for the mean observed allelic diversity per locus (*A*) and the effective number of alleles (*n*_*e*_) while NAV sample showed the lowest ones. In all populations, the average observed heterozygosity (*H*_*o*_) was lower than average expected heterozygosity (*H*_*e*_), mainly in NAV sample where a large homozygote excess was observed. The *F*_IS_ values, as expected, confirm the results (*F*_IS_ values, Table 3).

Significant deviations for Hardy-Weinberg expectations were found for Ap2 and Ap3 in all populations, as well as for Ap6 locus, excepting RIL sample. No significant linkage disequilibrium was detected between pairs of loci in these populations.

**Figure 4:**
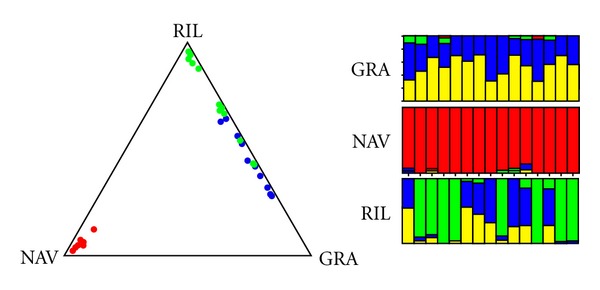
Summary of the clustering results for the *A. italicus* data assuming three populations. Each point shows the mean estimated ancestry for an individual in the sample. For a given individual, the values of the three coefficients in the ancestry vector *q*_(*i*)_ were given by the distances to each of the three sides of the equilateral triangle.

Clustering analysis by STRUCTURE (Figure 4) revealed that the three geographic groups (GRA, NAV, and RIL) indeed represented four genetically distinct populations.

**Figure 5:**
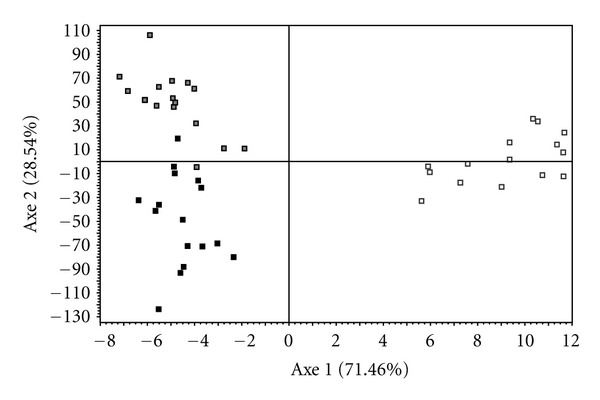
Correspondence analysis: projection of the individuals on the plane defined by the first two axes. Black, white, and grey dots codifying for GRA, NAV, and RIL populations, respectively.

The correspondence analysis (CA, Figure 5) agreed with STRUCTURE analysis. The first axe (70% of the inertia) clearly separated NAV sample from the remaining two studied populations, as the mtDNA markers did. The second axis (around 30% of the inertia) disjoined farmed population (RIL) from GRA, although a narrow area exists where individuals belonging to these two populations overlap.

According to Wilcoxon's signed ranks test, Rho_ST_ and *F*_ST_ values were similar (*P* > 0.05). The highest *F*_ST_ genetic distance was found between GRA and NAV samples (*F*_ST_ = 0.1030) and the lowest between GRA and RIL populations (*F*_ST_ = 0.0518).

## 4. Discussion

The main goal of the present work was the study of the genetic variability of three Spanish samples (Table 1)—located at distinct scenarios—of white-clawed crayfish, a threatened freshwater species in the Iberian Peninsula. Since the genetic diversity of a species reflects the influence of both historical and recent evolutionary events, a double approach was used to perform this task. On one hand, most phylogeographic studies of animals have relied on the analysis of mtDNA sequence variation due to its unique attributes and the different mutation rates compared to most nuclear genes. Its analysis has proven useful in defining major phylogeographic assemblages within species, including the European freshwater species—complex of *Austropotamobius* [37–39]. On the other hand, SSR nuclear loci have high mutation rates and tend to recover genetic variability quickly after the action of processes that affect it negatively. Thus, the molecular footprints on these SSR loci should be less long standing than in mitochondrial genome [40]. In this way, the SSR markers have been useful for addressing questions relating to current population structure of many freshwater species [41], including crayfish [42].

With respect to both analysed mtDNA sequences, *COI* gene resulted more sensitive for detecting genetic variability than *16S* rDNA. *COI* is a powerful marker for the study of the genetic variation at the intraspecific level in crayfish [43, 44] and other crustaceans [45–47] because its rate of molecular evolution is about threefold greater than that of *16S* rDNA gene [48]. Notwithstanding, given that the entire nonrecombining mitochondrial genome can be considered as a single locus from a genealogical perspective [49], the two mitochondrial markers—*COI* and *16S*—are discussed together in the present work.

Results indicate that the species exhibits in Spain a certain degree of genetic diversity at both mtDNA (*16S* rDNA and *COI* gene) and nuclear (SSR loci) level, despite the limited number of samples—from different scenarios—analysed in this study.

Four different mitochondrial haplotypes (Table 2) have been found, while for years the lack of genetic variability at mitochondrial level in Spanish populations of *A. italicus* was an accepted hypothesis [38, 50]. The degree of genetic diversity (Hd, *π*, Table 1) is higher than the previously reported for Iberian crayfish [37, 39] and similar, or even higher, to values obtained for others European populations with mitochondrial markers [40, 51]. Concerning microsatellite loci, four out of five markers tested were polymorphic, though most alleles showed low frequencies. The heterozygosity was relatively low albeit the mean number of alleles per locus detected was higher compared with other European populations [42, 52, 53] (Table 3, Figure 3). The existence of genetic variability in Spanish populations of this crayfish corroborates our former surveys, also at nuclear level, trough RAPD and ISSR markers [2, 11].

As shown in Figure 1, evidence for genetic differentiation among these three populations at mitochondrial level occurs. The distribution of mitochondrial haplotypes shows a clear geographic pattern (Figure 2) where Northern Spanish population do not share the haplotypes present in the Southern one, according to the reported by other authors [37, 39, 54]. The existence of fixed mtDNA differences between the populations analysed is consistent with severe restrictions on population size and geographic isolation. Given that mtDNA contains about one-fourth of the genetic variation included in the nuclear genome, large portions of the haplotypes can be wiped out during bottleneck events [55, 56].

Data from SSR analysis seem to bear out the above pattern. Although a certain degree of genetic variability was found, the four polymorphic SSR markers show a single allele nearly fixed (Figure 3) as expected for recently bottlenecked populations. Comparisons between Rho_ST_ and *F*_ST_ values indicate that genetic differentiation among these samples can be attributed to genetic drift. As shown in the STRUCTURE and correspondence analyses (Figures 4 and 5), the geographic labels matched very closely to the genetic clustering. The Northern population (NAV) is clearly separated from the other two. These analyses also highlight that some specimens from different populations are genetically similar at nuclear level, since some individuals from RIL sample are included in GRA cluster (Figure 4). The close relationship between GRA and RIL (hatchery) specimens could be explained by human translocations—a common practice in Spain since the 19th century—from GRA area to the source populations that gave rise to the current farmed sample or by the existence of a common ancestor population with a wider distribution in the far past.

Focusing on the Southern population, GRA shows a unique mitochondrial haplotype and a single allele per SSR locus at high or very high frequency, whereas the other ones remain at low frequencies (Table 3). The effective number of SSR alleles was also lower than expected, indicating a homozygote excess in the sample. The single haplotype found at mtDNA level suggest a strong bottleneck, proposed by some authors for this species [49, 50] around the last glaciation. In addition, SSR outcomes reveal a decline in population size recently. As a matter of fact, in the last two decades this population had suffered a drastic regression in number due to high mortalities caused by *Saprolegnia* spp., severe climatic droughts during the 1990s and a big flood at 1997 [57]. The genetic drift caused by bottlenecks was intensified, thus some haplotypes/alleles have been eventually lost while others became fixed according to its frequency [58]. Notwithstanding the presence of private SSR alleles is indicative of a recent increasing in GRA population size. The Andalusian Regional Government started at 2002 a conservation and management program of the white-clawed crayfish [59] that contemplates the development of emergency plans for drought and disease among other measures. Though the GRA sample is not directly situated in a protected area, possibly this population is benefiting from the strategies adopted in surrounding areas, including control/eradication of alien species.

The Northern population (NAV), although located in a protected area since 1996, showed the lowest variability at number of alleles per locus and exclusive alleles (Figure 3). These results indicate a recent and strong bottleneck since allelic diversity seems to be one of the most sensitive methods for detecting relatively recent demographic bottlenecks [13]. It is because rare alleles, which contribute little to the average heterozygosity, are easily lost during population size constrictions [58, 60]. In addition, the rate of inbreeding, unavoidable in small populations, is not negligible (*F*_IS_ = 0.8303, Table 3). Inbreeding reduces fitness [61] and usually enhances susceptibility to infectious diseases [62]. It is known that late in 20th century, high mortalities occurred in this region mainly due to the crayfish plague caused by *Aphanomyces astaci* [63]. Although two haplotypes were found, the mitochondrial analysis also supports the existence of a more ancient bottleneck because the 90% of crayfish shared the same mtDNA haplotype (Tables 1 and 2). NAV sample comes from a waterbody where *Pacifastacus leniusculus* also inhabits [64, 65] so, competition for space and diseases such as aphanomycosis could be some of the causes of the low genetic variability found in this population. Thus, genetic studies are necessary to ensure this species' future even in protected areas and to guarantee the existence of suitable levels of genetic variability in crayfish populations.

The hatchery population (RIL) exhibits the highest genetic diversity at mitochondrial level and moderate values for all the SSR variability parameters (Tables 1 and 3). The variability found may be explained by the fact that it was established in the 1980s with crayfishes from distinct basins and since then, it was kept under favourable conditions which have allowed to maintain a high population density [11, 66].

At present, Spanish populations of white-clawed crayfish are in regression due to environmental changes among other factors [2, 5, 67]. The current levels of genetic variability of *A. italicus* in Spain are affected by successive and drastic bottlenecks and consequently, by the action of the genetic drift, enhanced in these small and fragmented populations. However, ancient historical events such as population fragmentation, recolonizations from refugia during the ice ages [37, 38, 50, 68], or the formation of fluvial basins, must also have influenced their present structure, as well as it has been demonstrated in other species [69–71].

Our results underscore the usefulness of employing both mitochondrial and nuclear markers to assess current levels of genetic variability of the populations analysed as well as their genetic structure. Genetic information of the present study should be taken into account for future conservation plans. Though Spanish populations are in decline, a certain degree of genetic diversity has been detected. Given the pattern of the genetic variability found, it would be advisable an increase of within-population heterozygosity without eroding the differentiations that characterize the genetic structure of these Spanish samples. In this way, future works with more samples are needed in order to confirm these results and to provide guidelines about restocking purposes in each area. Hence, the crayfish hatchery analysed in this study (RIL) could be suitable for restocking the Southern population (GRA) but it is not fitted for the Northern one (NAV) that has to be considered as a different management unit [72], given its particular genetic characteristics.

## Figures and Tables

**Table 1:** Collection sites and genetic variability of *A. italicus* populations studied in the present work. Columns show, respectively, Code, Population, Watershed, Drainage Direction, and Collection sites they come from, H: hatchery. S: number of segregating sites; h: number of mtDNA haplotypes found; Hd and *π*: haplotype and nucleotide diversity, respectively. Last four columns refer to relative frequencies of haplotypes found in each population.

	Code	Population	Watershed/drainage direction	Collection sites	S	h	Hd	*π*	Relative frequencies			
									Hap_1	Hap_2	Hap_3	Hap_4
1	GRA	Brook Ermitas	Guadalquivir/Atlantic (West)	Albuñuelas/Granada	0	1	0	0	1	0	0	0
2	NAV	River Ega	Ebro/Mediterranean (East)	Estella/Navarra	1	2	0.2	0.00008	0	0.9	0	0.1
3	RIL	River Gallo	Crayfish hatchery, (H)	Guadalajara/ Castilla—La Mancha	1	2	0.556	0.0022	0.5	0	0.5	0

**Table 2:** Haplotypes found in *A. italicus* Spanish populations. Columns 2–5 refer to position of the SNP in the 2501 nt sequence. Bold numbers—first line—: SNP informative under parsimony. Freq: frequency in percentage of each haplotype in the total of individuals analysed. Num. Indiv.: number of individuals represented by each haplotype. Num. pop: number of populations in which they were detected.

	**531**	**1536**	2071	**2319**	Freq%	Num. Indiv	Num. Pop
Hap_1	C	T	A	A	50	15	2
Hap_2	C	C	A	A	30	9	1
Hap_3	T	C	C	G	16.67	5	1
Hap_4	T	C	A	G	3.33	1	1

**Table 3:** Genetic variation for five microsatellite loci in three Spanish populations of *A. italicus*. % HW: % loci in Hardy-Weinberg equilibrium; *A*: mean number of observed alleles; *n*_*e*_: effective number of alleles; *H*_*o*_: observed mean heterozygosity; *H*_*e*_: expected mean heterozygosity; *F*_*IS*_: inbreeding coefficient.

Population	Code	Parameter					
		% HW	*A*	*n*_*e*_	*H*_*o*_	*H*_*e*_	*F*_IS_
GRA	1	20	5.2000	2.5677	0.2133	0.4745	0.5591
NAV	2	20	3.2000	2.4785	0.0800	0.4579	0.8303
RIL	3	40	3.8000	2.5551	0.2267	0.4818	0.5383
